# Puricelli biconvex arthroplasty as an alternative for temporomandibular joint reconstruction: description of the technique and long-term case report

**DOI:** 10.1186/s13005-022-00331-4

**Published:** 2022-07-29

**Authors:** Edela Puricelli

**Affiliations:** Oral and Maxillofacial Surgery Unit/ Clinical Hospital of Porto Alegre (HCPA), School of Dentistry/Federal University of Rio Grande Do Sul (UFRGS), Rua Ramiro Barcelos, 2492, Porto Alegre, RS 90035-003 Brazil

**Keywords:** Temporomandibular joint reconstruction, TMJ ankylosis, Prostheses, Biomaterials, PMMA, Puricelli biconvex arthroplasty, Long-term follow-up

## Abstract

**Background:**

There are several indications for partial or total replacement of the temporomandibular joint (TMJ), including neoplasms and severe bone resorptions. In this regard, several techniques have been suggested to increase the functionality and longevity of these prosthetic devices. This case report describes the treatment of a TMJ ankylosis patient with the Puricelli biconvex arthroplasty (ABiP) technique, with a long-term follow-up.

**Case presentation:**

In 1978, a 33-year-old male polytraumatised patient developed painful symptoms in the right preauricular region, associated with restricted movement of the ipsilateral TMJ. Due to subcondylar fracture, an elastic maxillomandibular immobilisation (EMMI) was applied. Subsequently, the patient was referred for treatment when limitations of the interincisal opening (10 mm) and the presence of spontaneous pain that increased on palpation were confirmed. Imaging exams confirmed the fracture, with anteromedial displacement and bony ankylosis of the joint. Exeresis of the compromised tissues and their replacement through ABiP was indicated. The method uses conservative access (i.e., preauricular incision), partial resection of the ankylosed mass, and tissue replacement using two poly(methyl methacrylate) components, with minimal and stable contact between the convex surfaces. At the end of the procedure, joint stability and dental occlusion were tested. The patient showed significant improvement at the postoperative 6-month follow-up, with no pain and increased mouth opening range (30 mm). At the 43-year follow-up, no joint noises, pain or movement restrictions were reported (mouth opening of 36 mm). Imaging exams did not indicate tissue degeneration and showed the integrity of prosthetic components.

**Conclusions:**

The present case report indicates that ABiP enables joint movements of the TMJ, allowing the remission of signs and symptoms over more than 40 years of follow-up. These data suggest that this technique is a simple and effective alternative when there is an indication for TMJ reconstruction in adult patients with ankylosis.

## Background

The temporomandibular joint (TMJ) is classified as a complex, synovial, ginglymoarthrodial joint. It consists of the mandibular condyles and the mandibular fossa of the temporal bone, which are interposed by the articular disc. The mandible is stabilised and moved in different directions by a group of muscles. In particular, the mandible elevator muscles generate a resultant vector force in an anterosuperior direction [1].

The need for surgical removal of TMJ structures creates spaces between the jaw and the base of the skull, which can be reconstructed using cartilage tissue engineering [2] or alloplastic prostheses. Techniques for reconstructing anatomical structures of the joint, which are still under research, may include the condyle or the articular fossa alone or combine them into a total prosthesis [3–5].

Different biomaterials, such as chromium (Cr) and cobalt (Co) alloys, titanium, and ultra-high molecular weight polyethylene, can be used for prosthetic TMJ reconstruction [6, 7]. Another option is the use of poly(methyl methacrylate) (PMMA) bone cement, which is employed in several procedures such as prosthetic hip and knee fixation, cranioplasty [8, 9], vertebroplasty, and kyphoplasty [10]. Changes in the structure of bone cements have been introduced to improve their thermal, mechanical and biological performance [11, 12]. With new characteristics, multifunctional cements are efficient alternatives in the manufacture of prosthetic devices.

Prosthetic joints aim to decrease pain, morbidity, the need for reintervention, and high costs [3]. Thus, the ideal procedures for TMJ arthroplasties should be reliable and straightforward, with immediate mechanical resistance, long functional life, and stable fixation of the remaining bone structures [4, 13]. However, the durability of prosthetic devices is limited [5], since their materials do not have the remodelling capacity found in bone tissues, being more prone to natural wear. Alternatively, the lack of longevity of these prostheses may not be related to the resistance of the materials used, but to failures in reproducing the anatomical structures of the TMJ. The concave and convex shape of the TMJ surfaces causes a concentration of forces in the anterosuperior region [1]. However, the TMJ may have a favorable response to a change in the force vector [14, 15]. Therefore, alternative forms of prosthetic devices (e.g., biconvex) may be essential to disperse the vector forces exerted by the masticatory muscles [16, 17]. Puricelli biconvex arthroplasty (ABiP) proposes the use of two alloplastic, convex, juxtaposed, articulated surfaces for TMJ reconstruction [18–21]. In this way, the working contact between the components of the “new joint” is minimal, and friction is reduced. The present study aims to present details of the biconvex arthroplasty technique and reassess functional and radiographic parameters in a treated patient after long-term follow-up.

## Case report

**Fig. 1:**
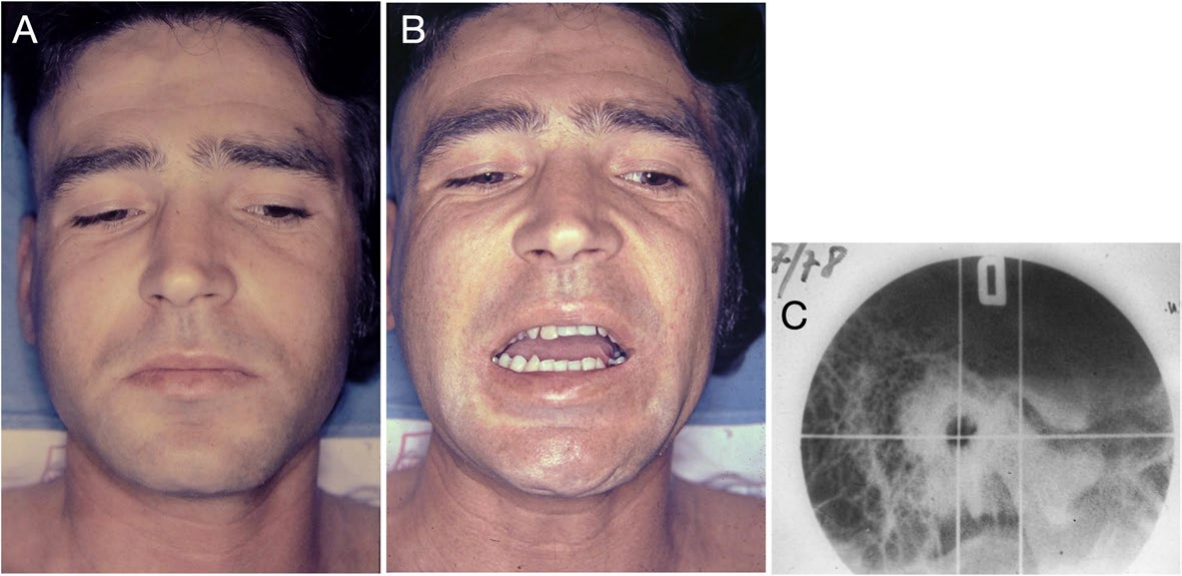
Preoperative facial aspect of the patient. **A** Facial symmetry and good lip sealing. **B** Maximum mouth opening (10 mm) with right side deviation (5 mm). **C** Linear tomography showing anterior displacement of the condylar segment; ankylosis area near the anterior wall of the auditory canal

A 33-year-old male patient was referred for ankylosis treatment of the right TMJ (Fig. 1a and b). Radiographic examinations revealed a fracture of the condylar process of the mandible (Fig. 1c).

## Past clinical history

In 1978, a polytraumatised patient was hospitalised for surgical treatments for lower limb fractures. Two weeks later, still in hospital, he complained of limitation of oral opening and pain in the right TMJ, confirming the condylar fracture. He was treated with maxillomandibular immobilisation for 14 days, according to his information.

After hospital discharge, the patient was referred for treatment of post-traumatic TMJ ankylosis. Six months after the accident, radiographic examinations revealed anterior displacement of the fractured segment and bone fusion (ankylosis) of this segment with the temporal bone on the right side (Fig. 1c).

## Description of the Puricelli biconvex arthroplasty

**Fig. 2:**
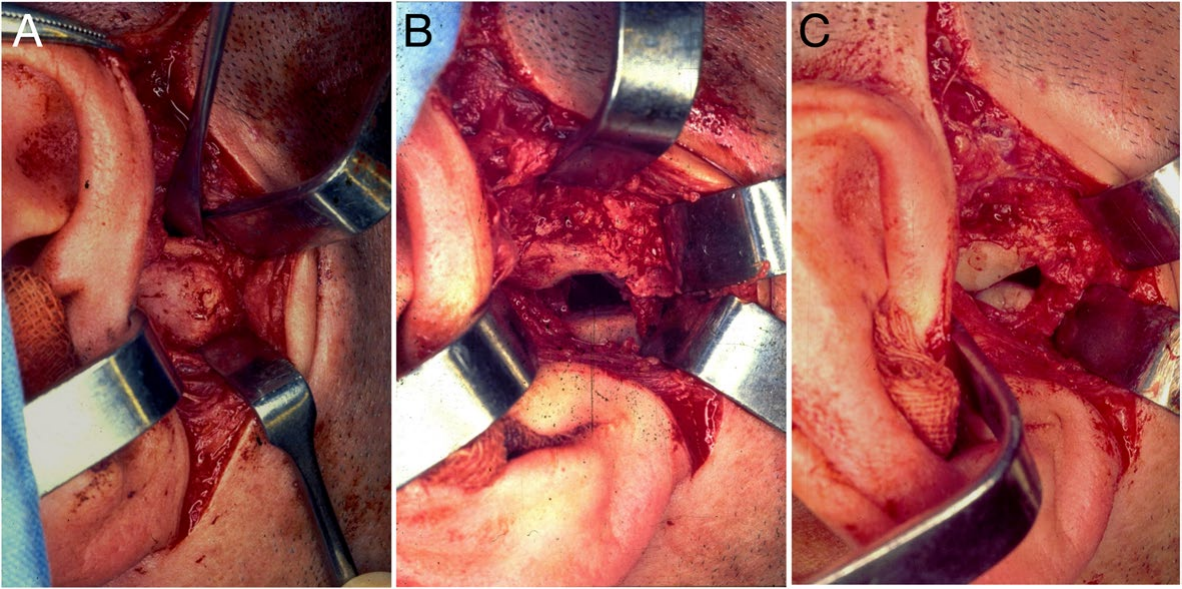
Intraoperative view of the Puricelli biconvex arthroplasty technique. **A** Preauricular incision with tissue detachment and exposure of the ankylotic mass. **B** Gap after removal of ankylotic mass and preparation of the extremity of the remaining condylar apophysis and glenoid fossa. **C** Visualisation of adapted poly(methyl methacrylate) (PMMA) components with minimal contact with each other

Besides preoperative routine examinations, the presence of other systemic or local bone diseases that may contraindicate the surgery should be investigated. Application of intermaxillary fixation devices is recommended in preoperative care for use in the intraoperative period. Fibreoptic nasotracheal intubation is performed for general anaesthesia in order to avoid tracheostomy due to the limitation of mandibular movements [22]. The affected TMJ is reached by a preauricular approach with temporal extension, followed by debridement and tissue detachment, both in the temporal and mandibular regions, for access to the ankylosed area. The joint capsule or any debris are removed. Less invasive muscle detachment allows surgical exposure of the pathological area, compatible with local manipulation (Fig. 2).

**Fig. 3:**
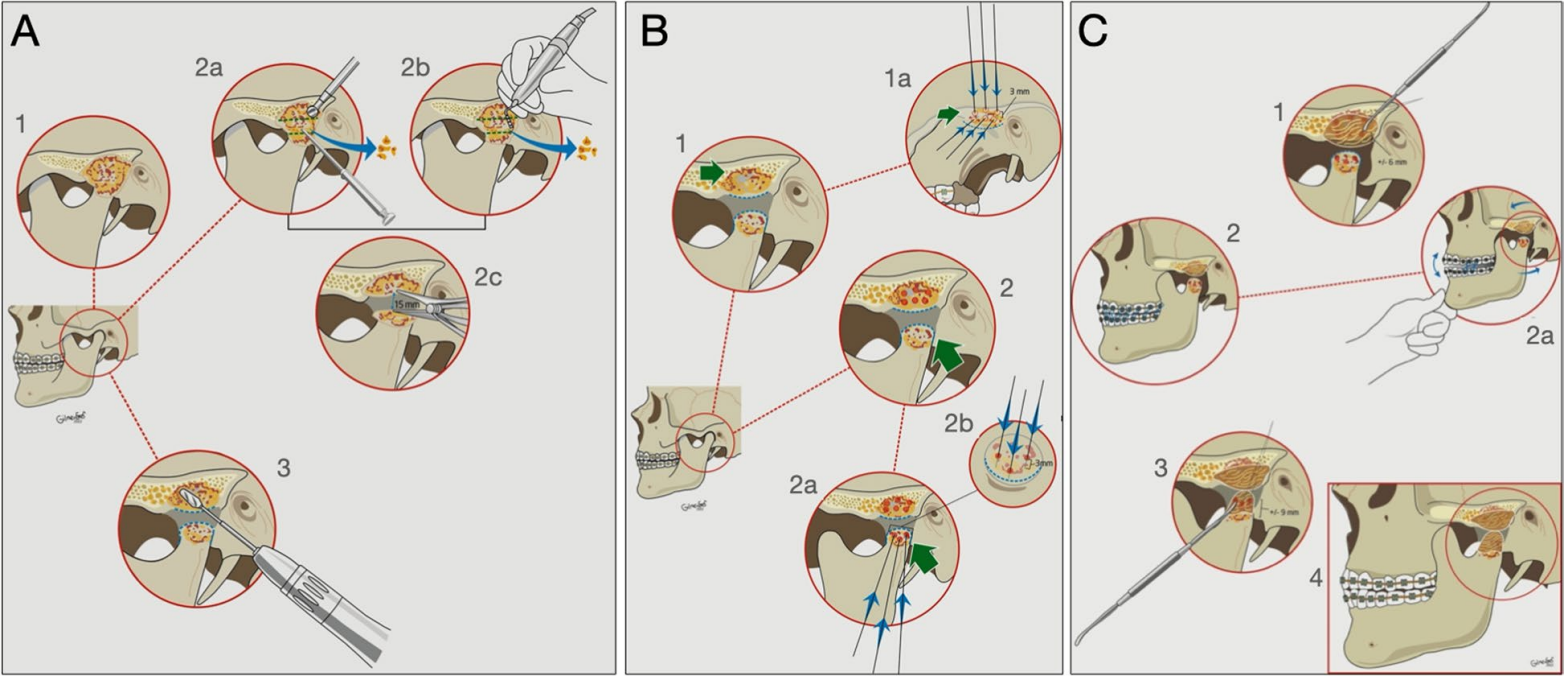
Puricelli biconvex arthroplasty. **A** Partial removal of ankylosed tissue and preparation of the areas for reconstruction. **1** TMJ ankylosis. **2** Removal of ankylosed mass. **2a** Perforation with spherical drill and ostectomy with chisel. **2b** Ostectomies using piezosurgery. **2c** Measurement of the gap using a surgical compass. **3** Sequential sculpting, with a milling cutter, of upper and lower (mandible stump) residual tissues in convex profiles. **B** Perforations of the sculpted remaining ankylosed area (upper and lower). **1** Perforations of the upper sculpted ankylosed mass (lateral view). **1a** Lateral view. **2** Perforations of the lower sculpted ankylosed mass (mandible stump). **2a** Lateral (lower) view. **2b** Upper view. Mean perforation depth is 3 mm. **C** Reconstruction of the remaining upper and lower ankylosed areas with PMMA. **1** Reconstruction of the upper structure with PMMA with overlay of the ankylosed area. The perforations are filled by mechanical pressing, and sculpted with a spatula into a convex structure with about 6 mm width, occupying part of the 15-mm gap. The perforations are filled with plastic PMMA by mechanical pressing, with total overlay of the upper structure. **2** An elastic maxillomandibular immobilisation (EMMI) is performed, for the correct positioning of the mandible in relation to the maxilla. **2a** With controlled oral lateral, and opening and closing movements, the best position for minimal contact after reconstruction of the mandible head is determined. **3** The mandible stump is filled with PMMA for reconstruction of a mandible head, sculpted using a spatula. The perforations are filled with plastic PMMA by mechanical pressing, with total overlay of the lower structure. **4** The EMMI is removed, and the minimal contact between the two structures results in successful restoration of joint function. The mandibular force vector now has an anteroposterior and inferosuperior direction in relation to the base of the skull

In the first phase of the surgery, partial resection of the ankylosed mass may be performed with surgical drills and chisel and/or piezosurgery. Any excess of soft and cartilaginous structures can be corrected by ablation. The ankylosed mass is included in the structures that will be reconstructed so that partial resection is performed from the centre to the edge. Tissue removal results in a vertical gap measuring on average 8 and 15 mm (in paediatric and adult patients, respectively). There are no indications for an ostectomy for anatomical reproduction of the glenoid cavity concavity. On the contrary, both in this and in the condylar region, residual tissues should be sculpted in convex profiles with a milling cutter (Fig. 3a).

In the case of longitudinal excess of the coronoid apophysis, diagnosed on preoperative computed tomography (CT) images, a uni/bilateral coronoidectomy is indicated. In cases of mandibular laterality correction due to ramus advancement, ipsilateral coronoidectomy can be indicated. The joint disc can be removed or not. In cases where it remains a mechanical barrier, the disc can be accommodated in parallel with the median contour of the mandible ramus, next to the excised condylar region.

In the second phase of the surgical procedure, having an upper and a lower slightly convex ankylosed structures, the upper structure is perforated. Using spherical drills with 1- or 2-mm diameter, 3- or 4-mm deep cortico-medullary or cortico-cortical perforations are made in the ankylosis remnants, which will mechanically retain the PMMA cement by micro-retention. The available local mass volume limits the number of these perforations, generally between three and five, for the preservation of the mechanical resistance of the region. In the temporal region, perforations follow the horizontal plane, similar to the application of screws in a prefabricated temporomandibular prosthesis associated with vertical perforations. After concluding with the upper structure, the lower structure (mandible stump) is perforated. The cortico-medullary and cortico-cortical horizontal and oblique perforations increase the micro-retentive surfaces and allow the construction of a hemisphere component ingrained in the previously sculpted residual stump (Fig. 3b).

The third surgical step corresponds to the reconstruction of the TMJ using PMMA (Surgical Simplex P Bone Cement, Howmedica International Inc, Limerick, Ireland). The remaining sculpted bone mass should be irrigated and aspirated to avoid tissue heating and obstruction of the perforations and medullary spaces with clots, debris, liquid and/or residues. Sequential reconstruction of the upper and lower regions of the TMJ is initiated by manipulating and inserting the plastic PMMA by mechanical pressing. The reconstruction process begins with an entire residual ankylosed upper area overlay with PMMA. This anterior–posterior axis component includes the space from the joint eminence to the limit anterior to the petrotympanic fissure. Vertically, its dimension must remain compatible with the joint space (the point of greatest convexity should reach, on average, 6 mm into the gap). Furthermore, its shape and position should allow the anteroposterior and inferosuperior support of the mandibular condyle. In case of a mandibular lateral deviation, this architecture adds more support and stability to the correction performed. During polymerisation of the PMMA, characterised by exothermic reactions and possible residual permanence of the monomer, constant irrigation and aspiration are maintained to avoid tissue damage.

After reconstructing the upper structure, the next step is to reconstruct the condyle on the mandibular stump (already slightly sculpted into a convex pattern and perforated). Usually, there is a restriction of mandibular movements in these patients. However, it is possible to obtain an acceptable partial dental intercuspation during the trans-operative period. In this “central position,” elastic intermaxillary immobilisation is performed. The joint space is maintained, allowing for the modelling of the arthroplasty. After confirming occlusal contacts and mandibular alignment with the midline, some elastic bands can be removed temporarily to provide mobility for mandibular manipulation during the reconstruction phase.

Maintaining the vertical dimension, the occlusion in maximum intercuspation, and respecting the space of the removed joint, PMMA is manipulated to create the new mandibular head. With the acrylic resin still in the plasticity phase, the PMMA is forced into the perforations by digital pressure. Concomitantly, the new condyle is moulded using a spatula, allowing for minimal contact with the upper structure. The mandibular force vector now has an anteroposterior and inferosuperior direction in relation to the base of the skull. Due to the risk of generating material residues and consequent inflammatory foreign body reactions, PMMA must not be worn out after polymerisation. After removing the EMMI, stability and dental occlusion are tested (Fig. 3c).

The surgical procedure ends with the placement of drains and sutures, which can be removed after 72 h and 7–10 days, respectively. Microporous tape is used post-operatively and is replaced during the first 21 days. Physiotherapy and speech therapy can start before the surgery and be continued postoperatively. For example, in the present surgical case, physical therapy was started 48 h before surgery and was maintained until the sixth postoperative month. In the first eight days, the exercises aimed to increase the maximum opening of the mouth and guide its closure. After that, laterality movements were progressively discouraged. Furthermore, proprioceptive exercises were gradually intensified according to the patient’s tolerance. Additionally, intramuscular pethidine hydrochloride (50 mg) was administrated 30 min before the exercises to prevent pain. If no pain was present after the first 24 h of exercise, the medication was discontinued. The patient was periodically revaluated during the postoperative period.

## Follow-up of the patient

**Fig. 4:**
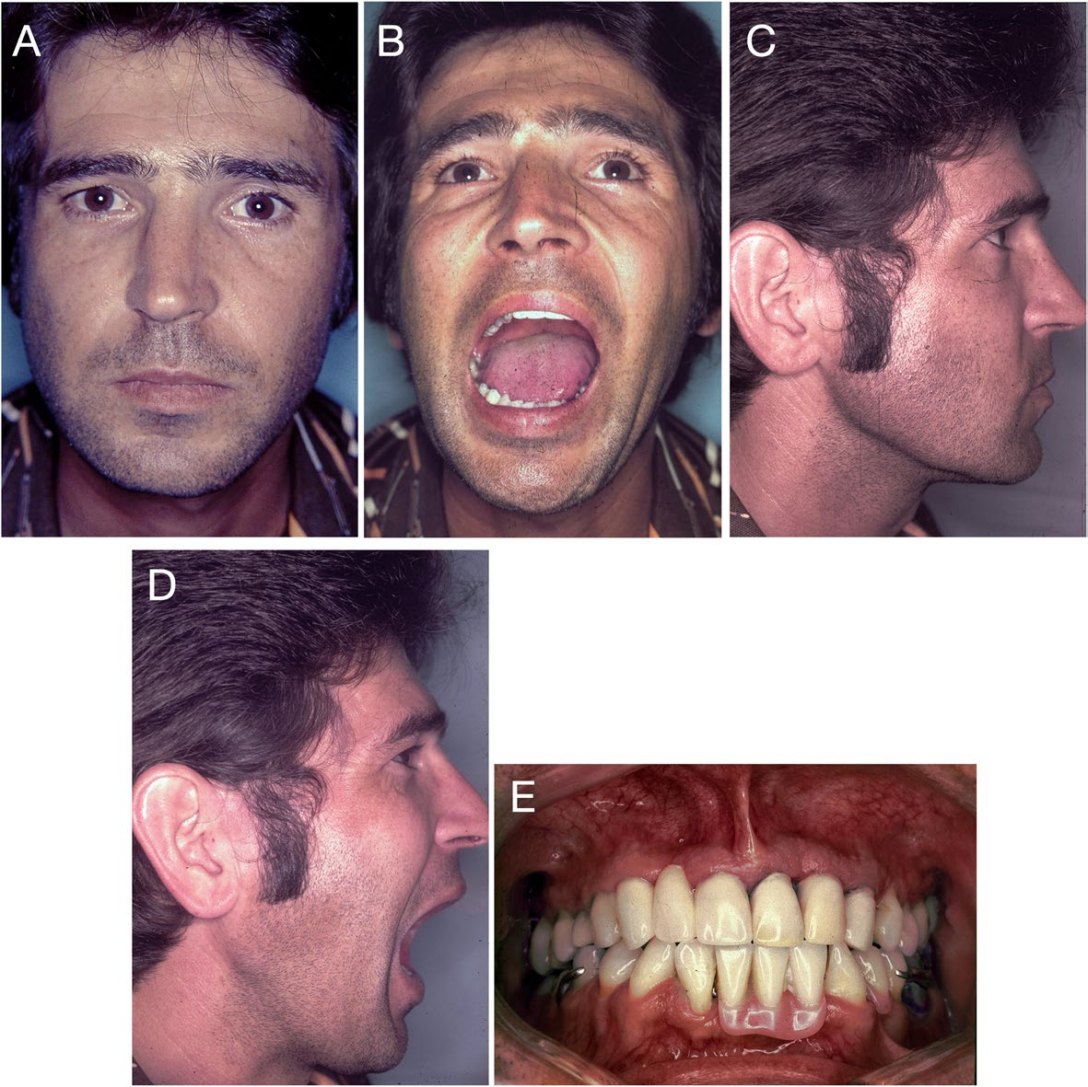
Postoperative facial aspect of the patient. **A** At the six-month evaluation. Facial symmetry as well as lip sealing are maintained. **B** Mouth opening (34 mm), deflection to the right side (4 mm). **C** Patient profile, right side. The scar of the preauricular incision is visible. **D** Maximum mouth opening. **E** Postoperative occlusion showing removable partial prostheses. Maintenance of masticatory and occlusal functions

**Fig. 5:**
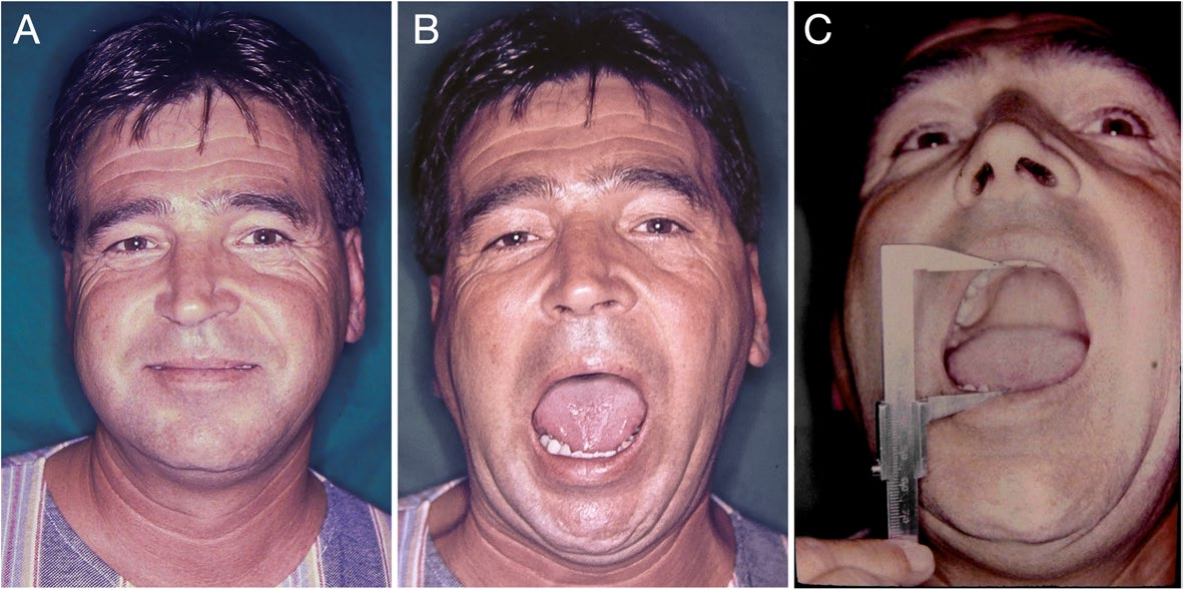
**A** Seventeen-year postoperative facial aspect (1978–1995). Maintenance of facial symmetry and lip sealing is observed. **B** Maximum mouth opening and right deviation (3 mm). **C** Measurement of maximum precision-opening of the mouth using manual callipers (36 mm)

The treatment results were followed up by measuring maximum mouth opening (interincisal distance), deflection in the opening, presence of noise, the intensity of spontaneous or palpation pain, and imaging tests. The patient was re-evaluated clinically at 6 months (1979), 17 years (1995), and 43 years (2021) after surgery. Furthermore, imaging exams were requested at the 6-month, 17-year and 39-year (2017) follow-ups. In addition to joint functionality, clinical evaluations addressed the signs and symptoms present. The Diagnostic Criteria for Temporomandibular Disorders (DC/TMD) questionnaire was used at the 43-year follow-up to standardise clinical findings and compare them with future evaluations. The postoperative condition progressively stabilised, and the 6-month follow-up showed a reduction in the signs and symptoms (pain and difficulty in joint movement) observed preoperatively (Table 1 and Figs. 4 and 5).

**Table 1:** Evaluation of mouth opening range. The interincisal distance and deflection to the right side were measured with the patient in maximum mouth opening

**Evaluation**	**Year**	**Mouth opening (mm)**	**Deflection in mouth opening (mm)**
Preoperative	1978	10	5
6-month follow-up	1979	34	4
17-year follow-up	1995	36	3
43-year follow-up	2021	36	3

## Joint pain and noise

Preoperatively, no joint noises were found, and the patient reported moderate to severe pain in the right TMJ region, which increased on palpation or movement. However, at the 6-month follow-up, no spontaneous or provoked pain or noise was present in the TMJ. Furthermore, this clinical condition remained stable when the patient was re-evaluated at 17 years. Forty-three years later, the patient reported no joint pain or headache episodes. Similarly, no joint noises were observed.

## Imaging results

**Fig. 6:**
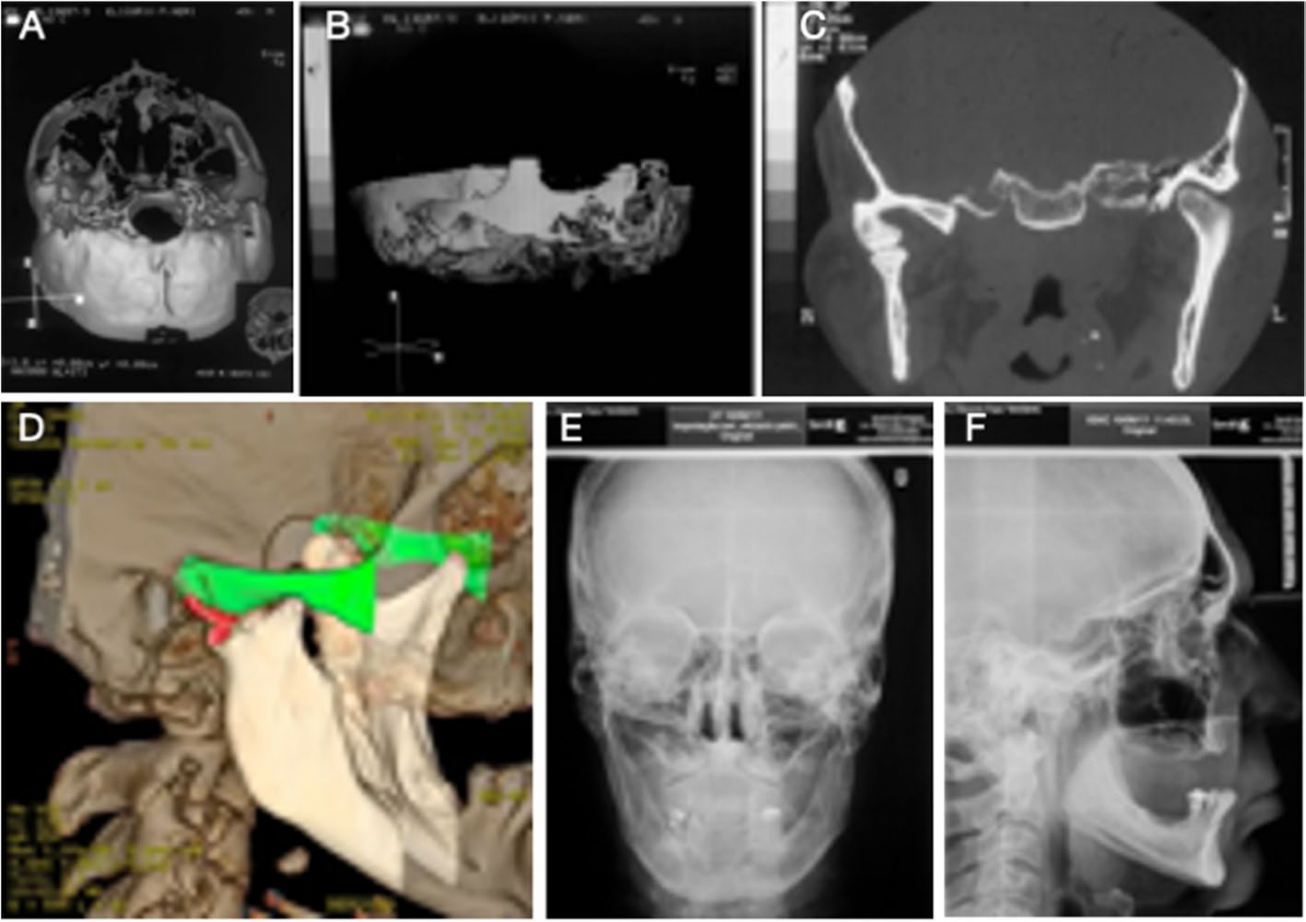
Postoperative imaging. **A**, **B** and **C** Computed tomography (17-year follow-up). The presence of poly(methyl methacrylate) (PMMA) components used in the Puricelli biconvex arthroplasty technique is observed on the right side. The components are stabilised in the initial position, near the base of the skull and the condylar region of the mandible. **D** Computed tomography with 3D reconstruction (39-year follow-up). Stability of the prosthetic components is observed, with no surgical reintervention during this period. **E** and **F** Frontal and lateral radiography, respectively (39-year follow-up). The facial skeletal relationship shows normal patterns

Preoperatively, a radiopaque area was observed in the right TMJ without delimitation between the upper and lower joint components. This image is compatible with the ankylosis process (Fig. 1c). At the 17- (Fig. 6a, b and c) and 39-year (Fig. 6d, e and f)follow-ups, imaging results showed the maintenance of the shape and position of the acrylic joint components. The contralateral TMJ maintained anatomical integrity (Fig. 6d). The different imaging modalities revealed stability of the prosthesis components near the base of the skull and the mandibular condyle region (Fig. 6d, e and f). At the 39-year follow-up, radiographic examinations demonstrated regular patterns of facial skeletal relationships (Fig. 6e and f).

## Joint movements

**Fig. 7:**
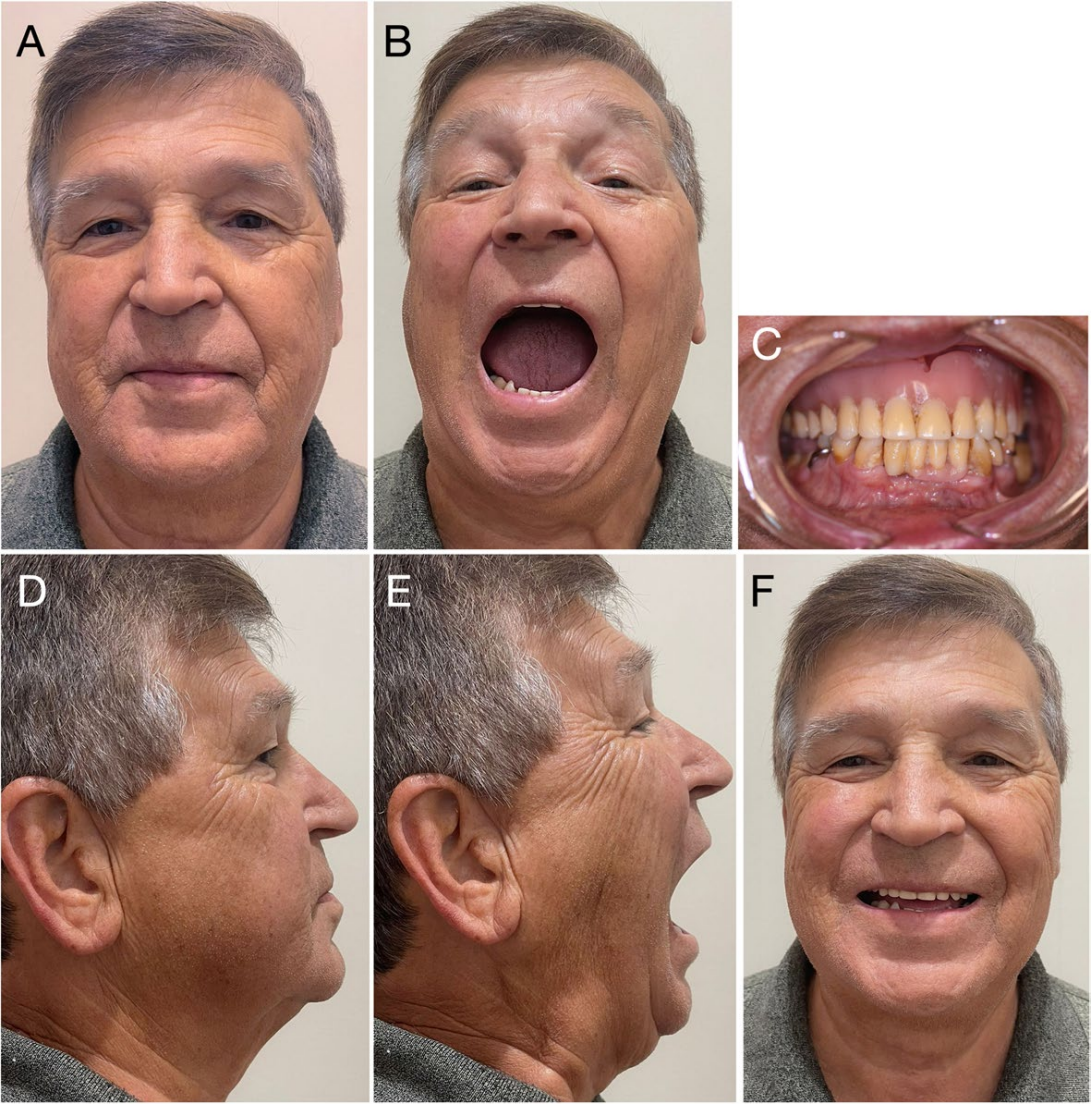
Postoperative facial aspect (43-year follow-up) with no surgical re-intervention in the period. **A** Maintenance of facial symmetry and lip sealing. **B** Maximum mouth opening (36 mm) and laterotrusion (3 mm). **C** Occlusion with total superior and partial inferior prosthetic rehabilitation, preserving masticatory function and without occlusal deviations. **D** and **E** Profile of the patient, with closed and open mouth, showing stability of the facial muscles. **F** Preserved facial expression

The interincisal distance, measured at maximum mouth opening, was evaluated at different follow-up times (Table 1). The preoperative evaluation showed severe restriction in mouth opening (10 mm), with 5 mm deflection to the right side (Fig. 1), suggesting an inability to perform complete rotational movement and minimal right condylar translation. However, at the 6-month follow-up, an improvement was observed in condylar movement, with the mouth opening increasing to 34 mm and deflection to the right side of 4 mm. Seventeen years after the procedure (Fig. 4), the patient presented a slight increase in mouth opening (36 mm) compared to the previous evaluation and a deflection to the operated side of 3 mm. At the 43-year follow-up evaluation, no changes were observed in the interincisal distance (36 mm) or deflection to the right side (3 mm), and the functional patterns remained stable since the 17-year follow-up. However, the patient had received a new total prosthesis, which made a direct comparison of the evaluations more difficult. Forty-three years after the procedure, with no surgical re-intervention in the period, the patient reported no restrictions of joint movements (mouth opening, closing or laterality) or function (chewing hard or consistent foods, talking, kissing or yawning) (Fig. 7).

## Discussion and conclusions

This clinical report presents a new surgical approach for TMJ reconstruction using a simple, low-cost, and relatively easy-to-perform method. Alongside these advantages, the main characteristic of ABiP is the longevity of results, which has been observed not only in the patient here described, but also in patients of different age groups treated by our team ([18, 21] and manuscript in preparation). Thus, implementing innovative protocols that can improve patients’ conditions over time, facilitate surgical techniques and reduce expenses should be encouraged. Trauma-induced TMJ ankylosis is similar to fracture healing [23, 24]. Histologically, ankylosis presents a remodelling bone callus [25] with a progressive fusion between the temporal bone and the mandibular condyle [26–28]. Since the bony mass is not a neoplasm, its excision can be programmed and limited [29]. The characteristics of the ABiP protocol allow the use of the temporal and condylar regions for fixation and stabilisation of joint implants, added to the thermal isolation provided by the local bone thickness. Furthermore, computed tomography imaging in axial, coronal and sagittal sections provides a safe interpretation of the bone areas to be treated and controlled.

Adverse effects of PMMA may be related to its constituent methyl methacrylate monomer (MMA). For example, bone cement implantation syndrome (BCIS) may result from the release of MMA into the bloodstream during prosthesis cementation [30–32]. Similarly, local mechanisms are related to the cytotoxic effects of MMA in smaller injured areas [33]. In biconvex arthroplasty, abundant intraoperative irrigation minimises these effects, adding to the benefit of local refrigeration in the exothermic phase.

Exposure of bone to exothermic reactions for more than 60 s, with temperatures between 40ºC and 120 °C, represents a potential risk of bone necrosis [34]. Therefore, a thickness of the PMMA mantle between 5 and 7 mm has been recommended for hip implant prosthesis [35]. Previous studies have concluded that the generation of temperatures between 56ºC and 60 °C is directly related to the volume of the mass used. Mercuri [36] observed that using a small volume of PMMA can minimise the exothermic reaction produced on-site. In the ABiP technique, the convex structures present an average radius of 4.0 and 7.0 mm in paediatric and adult patients, respectively, which is compatible with the previous study.

Most of the current biomaterials are well tolerated by the organism. In addition, these biomaterials maintain structural integrity, achieve mechanical stability in the bone, and are not colonised by microorganisms [7]. However, complications related to excessive wear of the materials and possible immune reactions remain a challenge, stimulating continued investigation [37–39].

Foreign body reaction is a common process when first-generation biomaterials such as metals, ceramics and polymers (e.g., PMMA) are implanted into biological tissues [6, 7]. The reaction to PMMA bulk has been described as a limited biological response, characterised by the production of a thin fibrous layer containing monocytes, macrophages and foreign body giant cells [40–45]. In our experience, the use of PMMA did not show those deleterious biological effects.

Clinical signs of pain, discomfort and dysfunction may reflect adverse effects related to foreign body reactions [46]. However, in the present case, the patient did not report any of the signs/symptoms mentioned, indicating the acceptance of the material by the patient’s tissue. In 1986, Masquelet developed a procedure based on inducing a foreign-body granulation membrane by inserting a PMMA cement spacer between the bone defect ends [47]. Furthermore, the induced membrane is similar to the periosteum or a pseudo-synovial tissue, having biological properties [48, 49]. Therefore, the technique can be used in significant diaphyseal defects. Moreover, this induced membrane is able to imitate the TMJ articular capsule. In the case reported here, results observed after 40 years confirm the biocompatibility of the PMMA cement and its applicability in the ABiP approach.

Simplex P cement has been on the market for more than 50 years. Cementation techniques using Simplex P are currently classified as first, second and third generation [7]. ABiP uses the first-generation technique, which involves manually mixing cement in a surgical bowl using a spatula. The preparation of the bone area is conservative, and part of the spongy bone is maintained in order to anchor the alloplastic material [4, 7]. Under digital pressure, the unpolymerised PMMA is inserted at an average depth of 3 to 5 mm. When the bone surface is cleaned and less hydrated, the depth of penetration of the material can increase, resulting in greater strength and mechanical resistance at the bone/polymer interface, through the pegged anchorage system concept.

After polymerisation, the PMMA cement maintains its shape, whether buried in the medullary space or carved into the external surface. Therefore, stable results can be achieved even during the intraoperative period. The glenoid fossa is altered from its concave to a convex shape in the biconvex joint configuration. The prosthetic cranial component is fixed on the lateral aspect of the glenoid cavity. Furthermore, the residual ankylosed mass supports the fixation of the PMMA joint unit, which is presented as a single hemispherical block of surgical cement. The mandibular condyle is constructed in the bone region, and the recommended maximum lower limit is at the level of the mandibular notch, with at least 1 mm of the bone surrounding the inlay rod.

For the condylar component, Xu et al. [50] proposed combining an association of the onlay plate with an inlay rod 3 mm in length, with a maximum diameter of 1.6 mm, a 10-degree taper angle, and at least 1 mm of surrounding bone. The authors suggest that using an inlay rod reinforces the connection between the prosthesis and the remaining bone tissue, as recommended in the ABiP approach. Ramos et al. [51–53] proposed a modification to the fixation of the condyle without plate and screws in the cortex of the mandibular ramus. According to the authors, the results of in vitro and ex vivo experiments suggest that the distribution of forces using intramedullary fixation of the condylar unit is similar to the intact condyle [51–53], a principle already applied in the ABiP technique. In the face of a restricted surface between the bone and PMMA, horizontal and oblique bone perforations enlarge the micro-retentive area.

In 1999, van Loon et al. proposed, for TMJ total prosthesis, a centre of rotation 15 mm inferior to the centre of the natural condyle, combined, if necessary, with a shift of up to 5 mm in the anterior direction [54], which is similar to the ABiP concept. In addition to providing a reference for the support of condyle movements, this cranial component allows an anteriorisation of the ramus for intra-operative corrections of vertical/lateral mandibular asymmetries, keeping this position stable. Furthermore, the interposed components used in ABiP have a relatively constant radius, creating a minimum contact area. This characteristic results in lower static friction, overcome by muscular kinetic friction [54]. Ackland et al. [55] emphasised the presence of redundant musculoskeletal systems with possible synergistic functions, where traction and compression provide stability and strength. According to Gallo et al. [17], although muscles produce only linear forces, the movements of the joints of the human body have, in almost all instances, a strong component of rotation, and can act with certain degrees of freedom. This kinetic component has also become evident during the joint function provided by ABiP. In addition, the physiology of the stomatognathic system provides stability between convex surfaces, which occur naturally during mandibular excursion movements [1].

In the TMJ, the force resulting from muscle activity forms an anterosuperior component [1]. Therefore, when there is a joint reconstruction by ABiP, the position of the upper component creates a new vector force component. Under normal conditions, for example, the condyle exerts reduced action force in the posterior region of the glenoid fossa. However, there is permanent posterior contact in ABiP, whether the joint is in motion or at rest (e.g., closed mouth).

The present proposal of more conservative surgery, with single surgical access, limited ostectomy, and maintenance of the residual bone structure in the mandibular ramus [18–21] is reinforced by Ramos and Mesnard [52], who suggest that this procedure allows surgical revisions if necessary. In addition, ABiP has other encouraging characteristics, such as low cost, easy execution and longevity, shown here for over four decades. New technologies (e.g., material, tissue and movement engineering) should improve the ABiP technique, stressing its use for TMJ reconstruction.

## Data Availability

Not applicable.

## Abbreviations

ABiP: Puricelli biconvex arthroplasty
TMJ: Temporomandibular joint
BCIS: Bone cement implantation syndrome
MMA: Methyl methacrylate
PMMA: Poly(methyl methacrylate)
